# Dispersibility and Storage Stability Optimization of High Dose Isoniazid Dry Powder Inhalation Formulations with L-Leucine or Trileucine

**DOI:** 10.3390/pharmaceutics12010024

**Published:** 2019-12-25

**Authors:** Imco Sibum, Paul Hagedoorn, Markus P. G. Kluitman, Martijn Kloezen, Henderik W. Frijlink, Floris Grasmeijer

**Affiliations:** Department of Pharmaceutical Technology and Biopharmacy, Faculty of Science and Engineering, University of Groningen, 9700 AB Groningen, The Netherlands

**Keywords:** high dose pulmonary delivery, dry powder inhaler, drug formulation, inhalation, tuberculosis

## Abstract

Tuberculosis is the leading cause of death from a single infectious pathogen worldwide. Lately, the targeted delivery of antibiotics to the lungs via inhalation has received increasing interest. In a previous article, we reported on the development of a spray-dried dry powder isoniazid formulation containing an L-leucine coating. It dispersed well but had poor physical stability. In this study, we aimed to improve the stability by improving the leucine coating. To this end, we optimized the spray-drying conditions, the excipient content, and the excipient itself. Using L-leucine, the tested excipient contents (up to 5%) did not result in a stable powder. Contrary to L-leucine, the stability attained with trileucine was satisfactory. Even when exposed to 75% relative humidity, the formulation was stable for at least three months. The optimal formulation contained 3% trileucine *w*/*w*. This formulation resulted in a maximum fine particle dose of 58.00 ± 2.56 mg when a nominal dose of 80 mg was dispersed from the Cyclops^®^ dry powder inhaler. The improved moisture protection and dispersibility obtained with trileucine are explained by its amorphous nature and a higher surface enrichment during drying. Dispersion efficiency of the device decreases at higher nominal doses.

## 1. Introduction

Tuberculosis (TB) is the leading cause of death from a single infectious pathogen worldwide [1]. It is a predominantly pulmonary disease which is transmitted by air [2]. During the infection, a cell-mediated immune response is developed, which results in the formation of poorly vascularized granulomas and tubercles [3,4,5]. In these granulomas and tubercles, TB is encapsulated and protected from anti-TB drugs. This leads to sub-bactericidal antibiotic concentrations at those sites, which introduces the risk of bacterial resistance development. Increasing the systemic dose, in order to increase the drug concentrations in these granulomas and tubercles, may lead to a higher incidence of adverse drug reactions. Therefore, a more targeted drug delivery that results in higher drug concentrations at the site of infection, such as pulmonary administration, could have substantial advantages [6]. As discussed in numerous papers, dry powder inhalers (DPIs) are suitable to administer the high dose anti-TB drugs to the lungs [3,6,7].

Isoniazid, together with rifampicin, is considered the most powerful anti-TB drug available [1,8]. In a previous article, we reported a spray-dried dry powder isoniazid formulation, which contained 5% L-leucine [9]. The formulation was well emitted from the Twincer DPI with a high fine particle fraction. However, the physical storage stability of the formulation was poor, as fusion between particles was observed after a mere two days. We assumed that this was the result of exposure to moisture, and that optimizing the leucine coating around the particles would improve the physical stability.

L-leucine has been known to provide moisture protection. Li et al. [10] hypothesized that this moisture protection resulted from the fact that L-leucine crystalizes during spray-drying and that these crystals enrich at the particle surface. The crystals have a low water uptake and thereby reduce the interaction between moisture in the air and the surfaces of the particles [10]. Whether a compound enriches at the surface during the spray-drying process and to what extent is described by the Peclet number. A Peclet number above one results in surface enrichment, while a number below one does not. The following formula is used to calculate the Peclet number: Pe = k/8D, where k is the evaporation rate, which depends on the settings of the spray drier, and D is the diffusion rate. The diffusion rate refers to the transport rate of either the molecules or the solid particles through the droplet; it therefore not only depends on the compound, but also on the state. A solid compound will have a lower diffusion rate than the same compound in solution [11]. It follows from the above that a change in process conditions, excipient content, or type of excipient affects the degree of surface enrichment attained during the spray-drying process.

In this paper, we set out to improve the enrichment of leucine at the surfaces of isoniazid particles in order to improve their physical storage stability with a minimal excipient content. To this end, we optimized the spray-drying conditions, the excipient content, and the excipient itself. We compared two excipients, L-leucine and trileucine. Trileucine consists of three L-leucine amino acids bound together with peptide bonds. The larger trimer molecule has a lower diffusivity than the L-leucine monomer. This lower diffusivity of trileucine may result in a higher degree of surface enrichment and, hence, better moisture protection.

## 2. Materials and Methods

### 2.1. Materials

Isoniazid and L-leucine used during this study were purchased from Sigma Aldrich (St. Louis, MO, United States). Trileucine was purchased from Bachem (Bubendorf, Switzerland).

### 2.2. Spray-Drying

To determine the optimal spray-drying (SD) conditions, isoniazid was spray-dried from 5% *w*/*w* L-leucine solutions in demineralized water. A B-290 mini spray drier, obtained from Büchi (Flawil, Switzerland), was used. The feed rate was controlled by an NE-300 syringe pump, obtained from ProSense B.V. (Oosterhout, the Netherlands). The inlet temperature and feed rate settings tested are shown in Table 1. Other conditions, such as an atomizing air flow of 50 mm, aspirator air flow of 100%, and a total solute concentration of 50 mg/mL, were kept constant. Demineralized water was used to prepare the samples in duplicate, which were spray-dried on separate days. Samples were analyzed the day after production.

### 2.3. Stability Study

**Figure 1:**
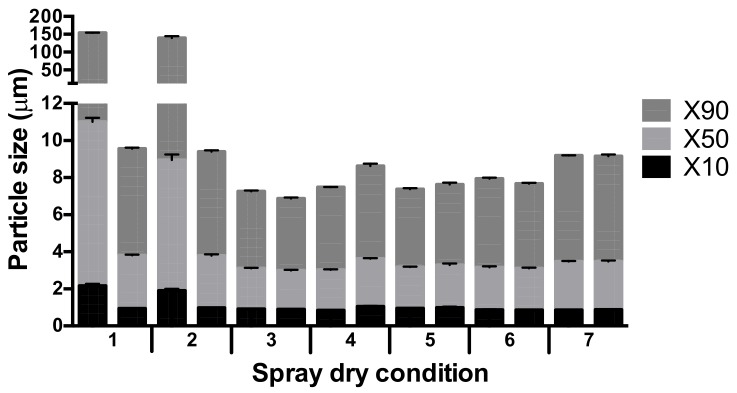
The primary particle size distributions resulting from the seven spray-drying conditions (see Table 1) for the isoniazid formulation with 5% L-leucine *w*/*w*. The duplicate samples are shown next to each other, for each sample *n* = 3, average ± standard deviation (SD) is displayed

Conditions 1 and 3 (Table 1) were used to determine the optimal excipient and excipient content with regard to physical storage stability and dispersibility. Condition 3 was used as it was deemed the most optimal (Figure 1). Condition 1, which has an inlet temperature of 40 °C instead of 120 °C, was added to determine if in the follow up experiments it performed poorer than condition 3, not only at the start but also over time. L-leucine and trileucine were tested as excipients in contents of 1, 2, 3, and 5% *w*/*w* for L-leucine and 1, 2, and 3% *w*/*w* for trileucine. Trileucine was not tested using the 5% *w*/*w* content because of its low solubility. The powders were stored in duplicate in desiccators at 0, 43.5, and 75% relative humidity (RH) at 30 °C in open containers. The 43.5% and 75% RHs were reached by the use of saturated solutions of potassium carbonate and sodium chloride, respectively, and 0% RH was reached by purging the desiccator with dry nitrogen. Samples were analyzed after one day, one week, one month, and, for the trileucine samples, also after three months. Samples that performed poorly at a certain time point were not followed up any further.

### 2.4. Primary Particle Size Distribution Analysis

The primary particle size distributions of the spray-dried formulations were measured by laser diffraction analysis. This size distribution is understood to represent the particle size distribution of the powder when dispersed to an aerosol free of agglomerates. So, this is the minimum size that can be obtained upon dispersion by the inhalers.

The analyses were performed with a HELOS diffraction unit equipped with the RODOS dry powder disperser, both obtained from Sympatec (Clausthal-Zellerfeld, Germany). Approximately 10 mg of dry powder was placed on the RODOS dry powder disperser prior to dispersion at 3 bars. The HELOS, fitted with an R3 lens, can size particles in a range of 0.1–175 μm. All measurements were performed in triplicate. The average and standard deviation was calculated over the three measurements, or over six when the two duplicate samples were grouped together.

### 2.5. Inhaler Dispersion Analysis

The particle size distributions in the aerosols from the Twincer^®^ and Cyclops^®^ inhalers [12,13], which likely include small agglomerates, were measured by use of the HELOS diffraction unit equipped with an INHALER2000 adaptor, provided by Sympatec (Clausthal-Zellerfeld, Germany) [14]. The HELOS, fitted with an R3 lens, can size particles in a range of 0.1–175 μm. Dry powder was weighted in a single dose cartridge, which was then placed in a Twincer^®^ or Cyclops^®^ inhaler. The inhaler was emptied by the INHALER2000 adaptor by means of a negative pressure, drawing the formed aerosol through the HELOS diffraction unit. For the stability study, measurements were performed at 4 kPa with a powder dose of 50 mg. All samples were measured in duplicate and the duplicate samples were grouped (i.e., *n* = 4). The fine particle fraction (FPF) was calculated as a percentage of the delivered dose. Retention of the powder by the inhaler was determined gravimetrically. For other dispersion measurements, one of the duplicate powders was measured in triplicate (*n* = 3). To determine the effect of the pressure drop, samples were analyzed at five different pressure drops, ranging from 2 to 6 kPa in steps of 1 kPa. To determine the effect of the dose on dispersion performance, four doses were tested in addition to 50 mg, namely, 75, 80, 90, and 100 mg. The fine particle dose was calculated multiplying the FPF with the emitted mass (= FPF * (1 – retention)).

### 2.6. Dynamic Vapor Sorption

The 1% and 3% trileucine formulations spray-dried at 40 °C and a 3% L-leucine formulation spray-dried at 120 °C were analyzed by dynamic vapor sorption (DVS). A DVS-1 (Surface Measurement Systems, Middlesex, United Kingdom) was used for the measurements. About 65 mg of sample was placed in the apparatus. The measurement was started at 0% RH and increased to the next RH level when the mass had stabilized, indicated by a mass change per time interval (dm/dt) <0.0005%/min. The RH was increased in steps of 10% to a maximum of 90% RH, after which the RH was decreased to 0% RH with the same step-size.

### 2.7. Time-of-Flight Secondary Ion Mass Spectrometry

The starting materials and three spray-dried samples, the 1% and 3% trileucine formulations spray-dried at 40 °C and a 3% L-leucine formulation spray-dried at 120 °C, were analyzed with time-of-flight secondary ion mass spectrometry (TOF-SIMS). An Ion-Tof TOF-SIMS IV (Münster, Germany) was used in negative and high-current bunched mode to obtain surface spectra. These spectra were taken with moderate lateral resolution (around 3 µm) and high mass resolution (ΔM/M > 5000). Powder was dispersed on double-sided tape and smeared out lightly, after which excess powder was blown away to obtain a monolayer of particles. Measurements were performed in duplicate. Ratios between leucine and isoniazid molecules at the surface of the particles of the spray-dried formulations were then calculated. TOF-SIMS data were reported as a mass spectrum with the number of counts per mass-to-charge ratio (M/z) value. The M/z value represents the mass of an ion divided by its charge. Molecules, such as leucine and isoniazid, are turned into ions by the mass spectrometer during analysis. Ratios of leucine and isoniazid molecules were calculated by dividing the counts of M/z values corresponding to L-leucine or trileucine by the counts of M/z values corresponding to isoniazid. For this, one trileucine molecule was counted as three L-leucine molecules to facilitate comparison. As a result of the high-energetic emission and ionization process during the measurement, clusters of isoniazid and leucine (without peptide bonds) were formed. These were included in the measurements as well.

### 2.8. Scanning Electron Microscopy

Scanning electron microscopy (SEM) was performed on the spray-dried powders using a JSM 6460 SEM, supplied by JEOL (Tokyo, Japan). After fixating the samples by use of double-sided carbon tape, 10 nm of gold was sputter-coated onto them by a JFC-1300 auto fine coater purged with argon gas. Imaging took place with an acceleration voltage of 10 kV, a spotsize of 25, a working distance of 10 mm, under high vacuum, and by use of the secondary electron detector.

## 3. Results

In Figure 1, the results of the primary size distributions of the powders containing 5% L-leucine produced using the seven spray-drying conditions are shown. Conditions 1 and 2 seem to result in a poorly reproducible formulation, where one of the samples has an X90 of around 150 µm and the other an X90 of around 10 µm. The other conditions seem to be more reproducible, with a possible exception of condition 4, where one sample results in slightly larger particle sizes. Overall, condition 3 seems the best, with the smallest X10, X50, and X90 values and only minor differences between the duplicates.

**Figure 2:**
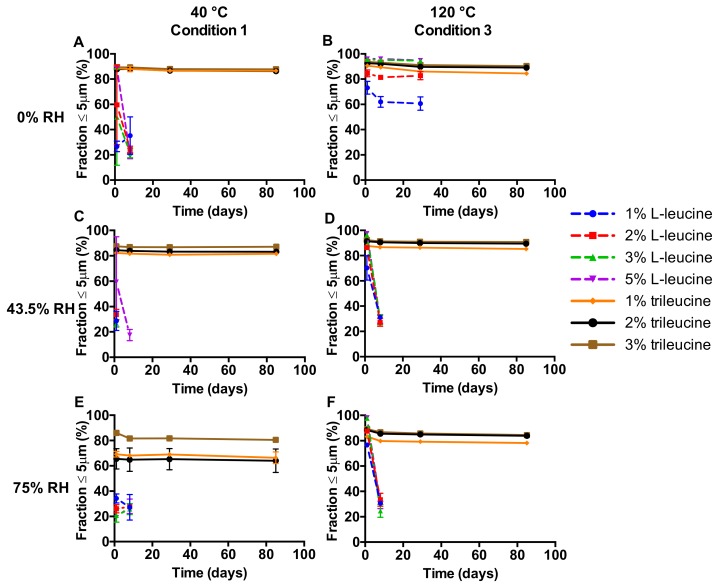
The fraction ≤5 µm of the primary particle size distributions at different storage relative humidity (RH) values over time (*n* = 6, average ± SD). Isoniazid formulations were spray-dried at two different inlet temperatures. Storage RH values are displayed on the left-hand side and spray-drying inlet temperatures on top.

Figure 2 displays the fraction ≤5 µm measured for the stability study powders. Powders containing either L-leucine or trileucine could both result in a powder suitable for inhalation, depending on the spray-drying settings, the excipient content, and the storage condition. For L-leucine, only the 3% and 5% *w*/*w* formulations spray-dried at 120 °C and stored under completely dry conditions were stable for at least one month. A higher relative humidity (RH), a lower inlet temperature, or a lower excipient content all resulted in powders that were not even stable for one week. The use of trileucine as an excipient results in considerably more stable formulations. All formulations, irrespective of inlet temperature, excipient content, and storage condition, resulted in powders suitable for pulmonary administration and they were physically stable for at least three months with almost no decrease seen in the fraction ≤5 µm.

**Figure 3:**
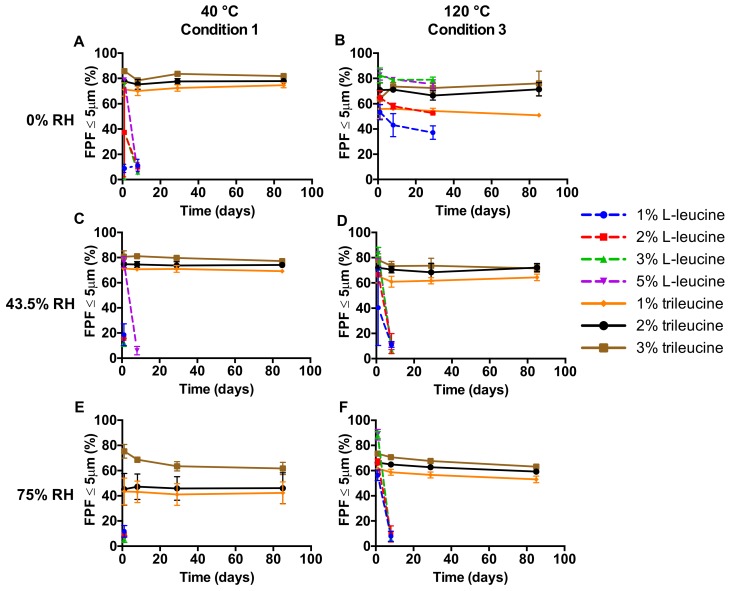
Fine particle fractions (FPFs) over time of isoniazid formulations dispersed from the Twincer^®^ dry powder inhaler (DPI) at 4 kPa (*n* = 4, average ± SD). Storage RH values are displayed on the left-hand side and spray-drying inlet temperatures on top.

The dispersion by the Twincer^®^ inhaler of the aforementioned powders is shown in Figure 3. As can be seen, for the L-leucine samples only the powders spray-dried at 120 °C, having an excipient content of 3% or 5%, and which were stored at 0% RH, resulted in an adequate FPF for at least one month. All other conditions tested resulted in adequate FPFs for less than one week. Again, the trileucine formulations performed considerably better. At all conditions tested, the powders resulted in FPFs suitable for pulmonary administration, even after storage for at least three months. The optimum formulation regarding FPF is the 3% trileucine formulation spray-dried at 40 °C. Stored at 0% and 43.5% RH, it results in a higher FPF than all other formulations over the three-month period. Only at 75% RH, the 3% trileucine formulation spray-dried at 120 °C disperses better than the one spray-dried at 40 °C. However, this difference is only 1.4% at the three-month time point. Furthermore, the formulation with 3% trileucine dried at 120 °C also shows a good performance.

**Figure 4:**
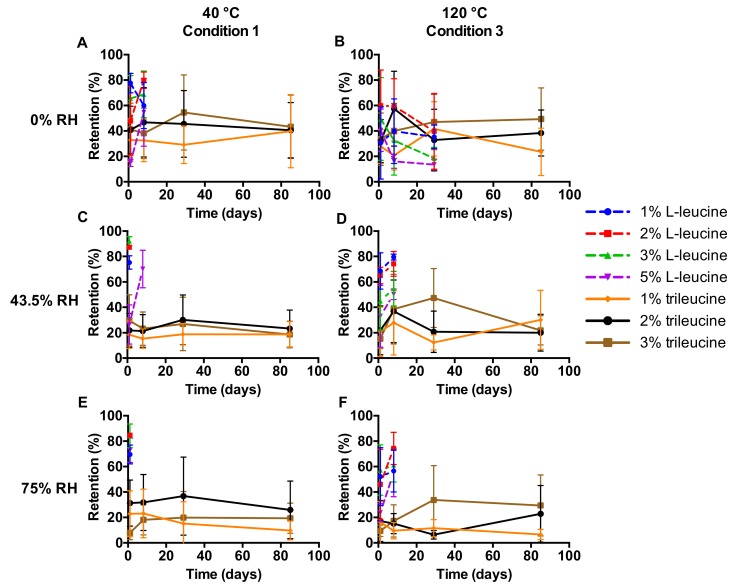
The powder mass retained in the Twincer^®^ as a function of storage time and expressed as a fraction of the total (nominal) dose (*n* = 4, average ± SD). Storage RH values are displayed at the top and spray-drying inlet temperatures on the left-hand side.

The powder mass retained in the inhaler during the dispersion measurements (i.e., “retention”) is presented in Figure 4. The retentions measured vary greatly and have substantial standard deviations. No apparent trend is visible except that the trileucine formulations generally result in a lower retention than the L-leucine formulations. Furthermore, storage of the trileucine samples at 0% RH result in a higher retention than storage at 43.5% and 75% RH. This difference may be caused by the stronger tribocharging at 0% RH.

**Figure 5:**
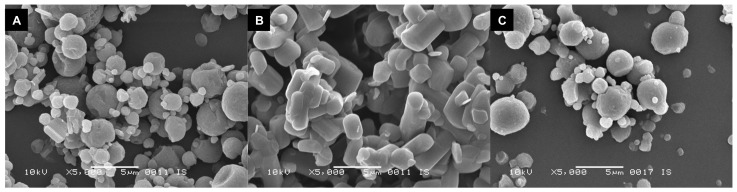
SEM images of spray-dried isoniazid. (**A**) shows the 3% L-leucine formulation spray-dried at 120 °C and stored at 0% RH for a month, while (**B**) shows the same formulation stored at 43.5% RH for a month. (**C**) shows the 3% trileucine formulation, spray-dried at 40 °C and stored at 43.5% RH for three months. Magnification: 5000x.

As can be seen in Figure 5, the 3% L-leucine formulation spray-dried at 120 °C retains its round morphology when stored under dry conditions for a month (Figure 5A). However, when the same formulation is stored under moist conditions (43.5% RH), the morphology changes substantially. The particles seem rod-like and fused together (Figure 5B). When the 3% trileucine formulation spray-dried at 40 °C is exposed to the same conditions for three months, it retains its round morphology and only very limited fusion can be seen (Figure 5C).

The results of the TOF-SIMS analyses on three spray-dried samples are shown in Table 2. The 3% trileucine formulation has a substantially higher leucine/isoniazid ratio at the surface compared to the 3% L-leucine formulation, showing that the lower diffusion rate of trileucine indeed results in a higher surface enrichment. Furthermore, the 3% trileucine formulation has a more than 3-fold higher leucine/isoniazid surface ratio than the 1% trileucine formulation.

**Figure 6:**
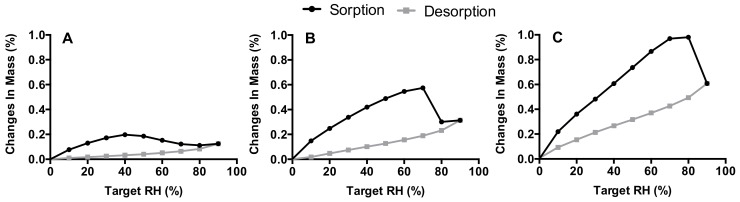
The dynamic vapor sorption (DVS) results. (**A**) shows the 3% trileucine formulation, spray-dried at 120 °C, (**B**) the 1% trileucine formulation, and (**C**) the 3% trileucine formulation. Both spray-dried at 40 °C.

The DVS results of spray-dried samples are shown in Figure 6. In Figure 6A, the 3% L-leucine sample increases in mass by only up to 0.2% at increasing relative humidity up to 40% RH during the sorption phase. After this, the mass decreases with a further increasing RH. This is likely the result of dissolution–crystallization, as discussed in our previous paper [9]. The threshold, when dissolution–crystallization occurs, is increased to 70% RH for the 1% trileucine formulation (Figure 6B) and to 80% for the 3% trileucine formulation spray-dried at 40 °C (Figure 6C). No water is taken up permanently for all of the samples as, during the desorption phase, they all return to 0% mass change. It is noteworthy that the maximum mass change appears to increase with increasing physical stability.

**Figure 7:**
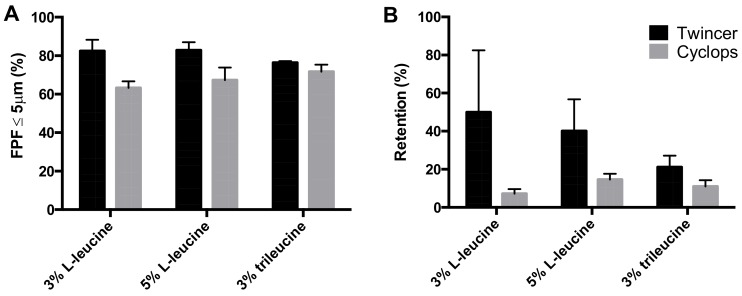
Comparison between the Twincer^®^ and Cyclops^®^ DPI with three isoniazid formulations dispersed at 4 kPa (*n* = 3, average ± SD). (**A**) shows the FPFs of the delivered dose from laser diffraction analysis, while (**B**) shows the retained powder mass as a fraction of the nominal dose (i.e., 50 mg). The L-leucine formulations are spray-dried at 120 °C, while the trileucine formulation is spray-dried at 40 °C.

Figure 7 shows a comparison between the Twincer^®^ and Cyclops^®^ dry powder inhalers tested with three formulations. As can be seen, the FPF for the Twincer^®^ is higher than for the Cyclops^®^ for all three formulations. However, powder retention by the Cyclops^®^ is reduced by 42.76%, 25.46%, and 10.20% for the 3% L-leucine, 5% L-leucine, and 3% trileucine formulations, respectively. Furthermore, powder retention is more reproducible with the Cyclops^®^, especially for the L-leucine formulations. However, even for the 3% trileucine formulation, where the difference between the two devices is minor, the Cyclops^®^ results in a 1.76 mg higher fine particle dose compared to Twincer^®^ when a nominal dose of 50 mg is dispersed.

**Figure 8:**
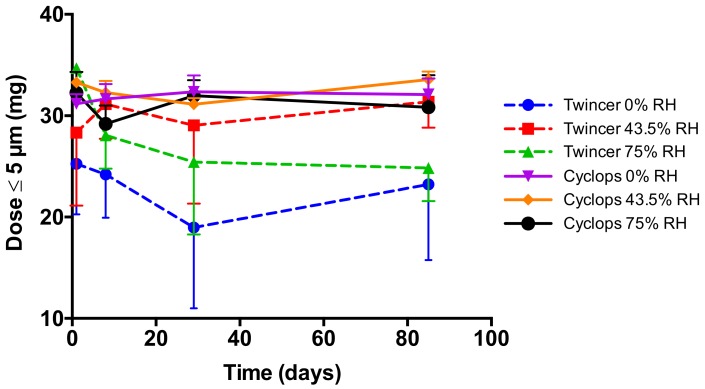
The fine particle dose delivered from the Twincer^®^ and Cyclops^®^ DPIs over a three-month storage period. Tests were performed with a 50 mg nominal dose of the 3% trileucine formulation spray-dried at 40 °C (*n* = 4, average ± SD). Powders were stored in open containers at 0, 43.5, and 75% RH.

The fact that the Cyclops^®^ delivers a higher fine particle dose of the 3% trileucine formulation compared with the Twincer^®^ is corroborated by Figure 8. At all storage conditions and at all time points tested, the Cyclops^®^ results in a higher fine particle dose that is also more reproducible. At all storage conditions, the fine particle dose was over 30 mg.

**Figure 9:**
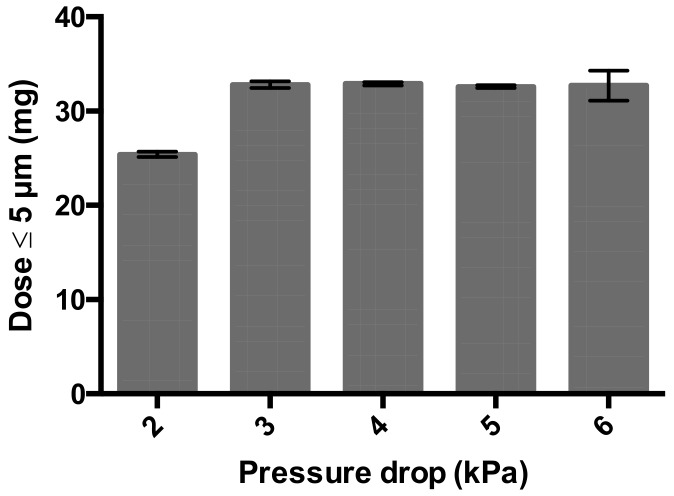
Fine particle dose from the Cyclops^®^ DPI loaded with 50 mg of the 3% trileucine formulation and dispersed at different pressure drops (*n* = 3, average ± SD). The fine particle dose is calculated by multiplying the FPF with the emitted mass (= FPF * (1 – retention)).

The robust dispersion performance of the cyclops with the 3% trileucine formulation is further demonstrated when the effect of the pressure drop on the fine particle dose from a 50 mg nominal dose is tested (Figure 9). At 2 kPa, the fine particle dose is 25.40 ± 0.27 mg. This increases to 32.80 ± 0.36 mg at 3 kPa and is stable at further increasing dispersion pressures.

**Figure 10:**
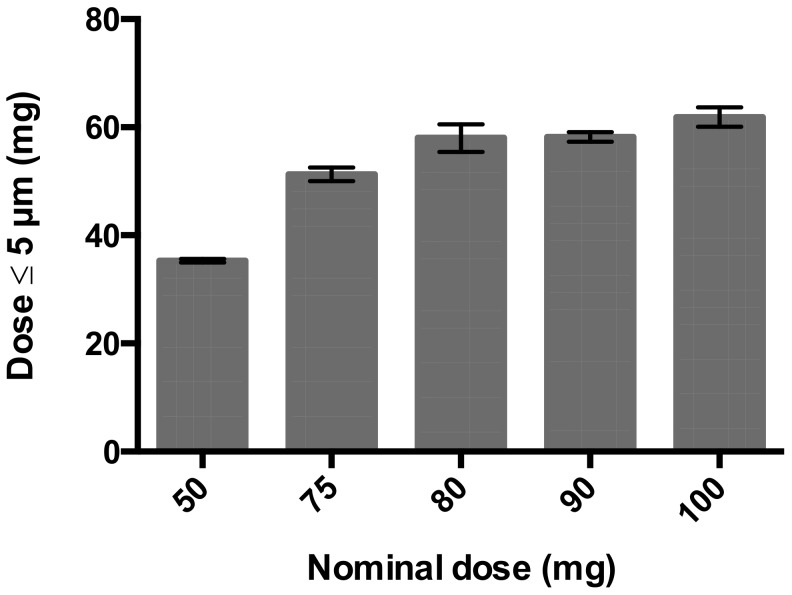
Fine particle dose from the Cyclops^®^ DPI loaded with different nominal doses of the 3% trileucine formulation and dispersed at a pressure drop of 4 kPa (*n* = 3, average ± SD). The nominal dose refers to the formulation (e.g., the nominal dose of 100 mg contains 97 mg isoniazid and 3 mg trileucine). The fine particle dose is calculated by multiplying the FPF with the emitted mass (= FPF * (1 – retention)).

Increasing the dose in the Cyclops^®^ DPI to doses over 50 mg substantially increases the fine particle dose for the 75 and 80 mg doses, as can be seen in Figure 10. However, at higher doses, there is hardly any further increase observed. Therefore, it is considered useless to increase the dose over 80 mg powder.

## 4. Discussion

The basic premise of this study was that coating of isoniazid particles is required to obtain a powder formulation that is suitable for pulmonary delivery using dry powder inhalation. Such a coating supposedly should prevent particle fusion during the spray-drying process, and also provide protection against aggregation caused by dissolution–crystallization during storage [9]. It follows that the best performing formulation in terms of primary particle size distribution, dispersibility, and storage stability is expected to result from those conditions that maximally favor surface enrichment of the coating excipient relative to isoniazid during the spray-drying process. Actually, the maximum surface enrichment of the excipient is expected to result ultimately in encapsulation of isoniazid.

An obvious approach to achieve encapsulation of isoniazid would be to increase the excipient content in the formulation. However, for L-leucine, it was shown that moisture protection of disodium cromoglycate was achieved only at excipient levels of at least 10–20% [10]. Since the isoniazid dose likely falls in the hundreds of milligrams range [9], such relatively high excipient contents are undesirable. Therefore, the simple approach of increasing excipient levels holds no practical relevance in formulating isoniazid for dry powder inhalation. Considering the findings of Li et al. [10], it is also not surprising that sufficient moisture protection of isoniazid was not achieved with L-leucine contents of up to 5% in this study (Figure 2 and Figure 5). Only when stored at 0% RH, an effect of L-leucine content on storage stability of the formulations spray-dried at 120 °C is observed (Figure 2). The excipient content has an effect on the encapsulation of isoniazid, as is shown in Table 2. With only a three times increase in trileucine content, the surface ratio increases roughly 12–15-fold, which may be indicative of a “Peclet effect” promoted by an earlier separation into the solid phase. The separation into a solid phase results in a decrease in diffusivity. Following Pe = k/8D, this increases the Peclet number and increases surface enrichment. The increased surface enrichment also has an effect on the moisture protection (Figure 6). The 3% trileucine formulation (Figure 6C) shows signs of dissolution–crystallization at higher humidities than the 1% formulation (Figure 6B). It is highly likely the same mechanism plays a role for L-leucine.

The inlet temperature and feed rate of the solution during spray-drying appears to affect the encapsulation efficiency of the excipients used, as for the L-leucine formulation, a reproducible fine particle size distribution was obtained only at inlet temperatures at or above 120 °C (Figure 1). Also, the stability of the L-leucine formulations at 0% RH was notably better after spray-drying at 120 °C (condition 3) than at 40 °C (condition 1) (Figure 2). However, the exact mechanism behind these results is unclear. At lower feed rates and higher inlet temperatures, higher evaporation rates of the solvent are attained. Following Pe = k/8D, this affects the Peclet numbers of the excipient and isoniazid to the same extent, and therefore, a substantial effect on encapsulation is not necessarily to be expected. A possible exception may exist if at 120 °C, L-leucine has a Peclet number above one, signifying surface enrichment, while the same is not true at 40 °C. However, in the literature, it is hypothesized that the formation of an L-leucine coating is dependent on the fact that L-leucine readily crystallizes when it reaches its saturation concentration during drying [10]. These crystals have a lower diffusivity, which increases the Peclet number. An increase in evaporation rate does shorten the time to supersaturation for L-leucine. However, it does the same for isoniazid. In fact, when calculating the time to saturation for isoniazid an L-leucine under conditions 1 and 3, isoniazid reaches supersaturation first at both conditions and at all excipient contents. The fact that L-leucine is enriched at the surface, which is shown in Table 2, might then be explained by a difference in rate of separation into the solid phase. While it is known that L-leucine readily separates into a solid when it reaches its supersaturation concentration in the form of crystals, for isoniazid it is not. In our previous paper, we showed that isoniazid is amorphous when collected in the spray drier, and then it quickly crystallizes [9]. However, the rate at which it turns into a solid during drying is unknown. If this is low, the L-leucine crystals may selectively enrich at the surface. It is unclear if a higher evaporation rate has an effect on this mechanism and/or how this explains the results shown in Figure 1 and Figure 2. Another mechanism that may explain these results is the fact that L-leucine is somewhat surface active [15]. For trileucine, which has a higher surface activity, it has been shown that this may have an effect on the surface enrichment [11,16]. Furthermore, for trileucine, the surface activity seems to increase with increasing temperature [17]. The extent to which this mechanism plays a role for L-leucine is not known. Nevertheless, although adjustment of the spray-drying conditions did result in satisfactory primary particle size distributions at a relatively low L-leucine content of 5%, adequate storage stability was not obtained (Figure 2). Considering the stability of the 3% and 5% L-leucine formulations at 0% RH, the reduction in fraction ≤5 µm does appear to be moisture-dependent, as would be the case with dissolution–crystallization.

Contrary to L-leucine, the physical storage stability attained with trileucine is very satisfactory, but conclusions regarding the exact mechanism of this effect cannot be irrefutably drawn. The improved storage stability with trileucine may result from a higher encapsulation efficiency. Trileucine has a higher molecular weight than L-leucine (358 versus 131 g/mol), a lower diffusivity, and a lower solubility (22 mg/mL versus 8 mg/mL), which results in a higher Peclet number [11,16]. Possibly even more so because of earlier separation into the solid phase. Surface enrichment is higher for trileucine than L-leucine with the same excipient content (Table 2). However, it is remarkable in this regard that 1% trileucine spray-dried at 40 °C results in a notably higher physical storage stability than 3% L-leucine spray-dried at 120 °C (Figure 2) with roughly 0.1–0.2 times the leucine to isoniazid surface ratio (Table 2)—a fact that is corroborated in Figure 6. The 1% trileucine formulation shows signs of dissolution–crystallization at a substantially higher RH than the L-leucine formulation. This suggests that other mechanisms than encapsulation are in play. L-leucine is known to crystalize during the spray-drying process, while trileucine separates in the solid state in the amorphous form [18]. Thus, L-leucine likely forms a crystalline coating and trileucine an amorphous one. Amorphous materials are usually more hygroscopic than their crystalline counterparts, possibly explaining why the trileucine samples absorb more water than the L-leucine sample in Figure 6. However, the amorphous coating does seem to be better at preventing, or delaying, diffusion–crystallization compared to crystalline L-leucine (Figure 5). So, while the extent of the surface enrichment plays a role in the physical stability, the exact mechanism as to why trileucine outperforms L-leucine remains unclear.

This study shows that formulation design and inhaler design go hand in hand in the development of dry powder inhalation products. Even though a physically stable powder with a primary particles size distribution suitable for pulmonary administration (Figure 2) and sufficient dispersibility from the Twincer was obtained (Figure 3), powder emission was poor (Figure 4). Perhaps this could have been improved by further optimizing the formulation, but this would have likely increased the excipient content. Instead, a different inhaler design, i.e., the Cyclops^®^, improved dose emission and reproducibility of the same formulation without losing dispersion performance (Figure 7 and Figure 8). Ideally, the inhalation flow rate dependence of the fine particle dose would be more pronounced than is attained in this study (Figure 9), as this theoretically results in a more flow rate-independent lung deposition [19]. The 3% trileucine formulation dispersed too well in that regard. This formulation and device combination is capable of dispersing a maximum nominal dose of 80 mg, administering a fine particle dose of 58.00 ± 2.56 mg (Figure 10). Increasing the dose to levels over 80 mg is not considered useful, since this hardly increases the lung dose. At 50 mg, the fine particle dose makes up 70.6% of the nominal dose; at 100 mg, this is only 61.9%. Higher doses are therefore only expected to increase throat deposition, which may induce a cough reaction and lower patient adherence. A dose system that does not release all the powder at once to the classifier but delivers the powder gradually might improve the efficiency and increase the fine particle dose. However, it has to be kept in mind that to deliver the particles deep in the lung, only a short time frame at the start of the inhalation is available where all the powder has to be delivered. In general, to be able to transport the particles to the lower airways, the powder has to be discharged in the first 0.5–1.5 L of air [7]. An optimum has to be found in that regard.

The applicability of the technology described in this study to other APIs is manifold. As discussed above, encapsulation of these APIs by trileucine may be achieved if they either have a higher solubility (8 mg/mL), a higher diffusivity, or separate into the solid phase slower than trileucine when dissolved in water. Further, their Peclet number should be lower than that of trileucine during the spray-drying process. The technology may be especially of benefit for other high dose pulmonary drugs, which, because of their physico-chemical properties, show poor dispersion behavior. In this respect, drug substances which suffer from a high hygroscopicity or deviating crystallization behavior are obvious examples, but also drugs that suffer from a poor stability can be considered.

As described in our recent review, high dose pulmonary drugs are receiving increasing attention—not only for the pulmonary administration of antibiotic drugs against TB, but also antibiotic drugs for cystic fibrosis treatment, anti-fungal drugs, chemotherapeutics for the treatment of lung cancers, pulmonary arterial hypertension drugs, and others [6]. As a result from the relatively high doses required for these applications (≥2.5 mg), formulation strategies such as adhesive mixtures are unsuitable. These drugs may benefit from co-spray-drying with trileucine to increase their dispersability and/or provide moisture protection. For example, aminoglycoside antibiotic drugs, which are hygroscopic [13], may benefit from the moisture protection provided by trileucine. In this study, we determined the dispersion by the inhaler as the primary indicator for the physical quality of the powder. However, in future studies, one could consider to determine powder properties that are easier to measure, such as density and flowability, since such properties may be used as an indirect indication of physical formulation stability and performance.

## 5. Conclusions

This study set out to improve the leucine enrichment at the surface of isoniazid particles in order to improve their physical storage stability using a minimal excipient content. L-leucine does not result in adequate moisture protection at any of the excipient contents tested. Higher excipient contents likely would have resulted in moisture protection. However, these high excipient contents are undesirable for high dose drugs. Optimizing the spray-drying conditions does result in satisfactory primary particle size distributions with relatively low L-leucine contents, but moisture protection is insufficient. Storage stability and moisture protection from trileucine-containing formulations are adequate at the 3% trileucine level, which has the highest leucine surface enrichment measured. This optimum formulation found is stable for at least three months, even when exposed to 75% RH. The mixture of isoniazid with trileucine 3% *w*/*w* is spray-dried at 40 °C and performs best when dispersed from the Cyclops^®^ DPI. The Cyclops^®^ is capable of dispersing a nominal dose of 80 mg of this formulation, resulting in a 58.00 ± 2.56 mg fine particle dose. These findings could contribute to the development of an inhalable isoniazid product, which may target the primary infection site of tuberculosis, possible eradicating mycobacteria considered resistant. In general, the understanding of the described mechanisms may contribute to achieve encapsulation and moisture protection for other APIs with low leucine excipient content.

## Figures and Tables

**Table 1:** Conditions used to determine the optimal SD parameters. The atomizing airflow, aspirator airflow, and final concentration in the feed solution were all kept constant.

Condition	Inlet Temperature (°C)	Feed Rate (mL/min)
1	40	1
2	60	2.5
3	120	1
4	140	4
5	160	2.5
6	160	7
7	160	12.5

**Table 2:** The leucine/isoniazid surface ratios found during the TOF-SIMS analyses. The L-leucine formulation is spray-dried at 120 °C, while the trileucine formulations are spray-dried at 40 °C (*n* = 2, both values shown).

Formulation:	3% L-Leucine	1% Trileucine	3% Trileucine
**Ratio:**	11.22–28.15	2.49–2.53	29.09–37.76

## References

[1] World Health Organisation (2018). Global Tuberculosis Report 2018.

[2] Pandey R., Khuller G.K. (2005). Antitubercular inhaled therapy: Opportunities, progress and challenges. J. Antimicrob. Chemother..

[3] Momin M.A.M., Tucker I.G., Das S.C. (2018). High dose dry powder inhalers to overcome the challenges of tuberculosis treatment. Int. J. Pharm..

[4] Ulrichs T., Kaufmann S.H.E. (2006). New insights into the function of granulomas in human tuberculosis. J. Pathol..

[5] Das S., Tucker I., Stewart P. (2015). Inhaled Dry Powder Formulations for Treating Tuberculosis. Curr. Drug Deliv..

[6] Sibum I., Hagedoorn P., de Boer A.H., Frijlink H.W., Grasmeijer F. (2018). Challenges for pulmonary delivery of high powder doses. Int. J. Pharm..

[7] Hoppentocht M., Hagedoorn P., Frijlink H.W., de Boer A.H. (2014). Technological and practical challenges of dry powder inhalers and formulations. Adv. Drug Deliv. Rev..

[8] Mubiligi J.M., Mpunga T., Tapela N., Okao P., Harries A.D., Edginton M.E., Driscoll C., Mugabo L., Riviello R., Shulman L.N. (2014). Drug-resistant tuberculosis in Eastern Europe: Challenges and ways forward. Public Health Action.

[9] Sibum I., Hagedoorn P., Frijlink H.W., Grasmeijer F. (2019). Characterization and Formulation of Isoniazid for High-Dose Dry Powder Inhalation. Pharmaceutics.

[10] Li L., Sun S., Parumasivam T., Denman J.A., Gengenbach T., Tang P., Mao S., Chan H.-K. (2016). L-Leucine as an excipient against moisture on in vitro aerosolization performances of highly hygroscopic spray-dried powders. Eur. J. Pharm. Biopharm..

[11] Vehring R. (2008). Pharmaceutical particle engineering via spray drying. Pharm. Res..

[12] De Boer A.H., Hagedoorn P., Westerman E.M., Le Brun P.P.H., Heijerman H.G.M., Frijlink H.W. (2006). Design and in vitro performance testing of multiple air classifier technology in a new disposable inhaler concept (Twincer^®^) for high powder doses. Eur. J. Pharm. Sci..

[13] Hoppentocht M., Akkerman O.W., Hagedoorn P., Frijlink H.W., De Boer A.H. (2015). The Cyclops for pulmonary delivery of aminoglycosides; A new member of the Twincer family. Eur. J. Pharm. Biopharm..

[14] De Boer A.H., Gjaltema D., Hagedoorn P., Frijlink H.W. (2002). Characterization of inhalation aerosols: A critical evaluation of cascade impactor analysis and laser diffraction technique. Int. J. Pharm..

[15] Gliński J., Chavepeyer G., Platten J.K. (2000). Surface properties of aqueous solutions of L-leucine. Biophys. Chem..

[16] Vehring R., Foss W.R., Lechuga-Ballesteros D. (2007). Particle formation in spray drying. J. Aerosol Sci..

[17] Lechuga-Ballesteros D., Charan C., Stults C.L.M., Stevenson C.L., Miller D.P., Vehring R., Tep V., Kuo M.C. (2008). Trileucine improves aerosol performance and stability of spray-dried powders for inhalation. J. Pharm. Sci..

[18] Wang H., Nobes D.S., Vehring R. (2019). Particle Surface Roughness Improves Colloidal Stability of Pressurized Pharmaceutical Suspensions. Pharm. Res..

[19] Demoly P., Hagedoorn P., de Boer A.H., Frijlink H.W. (2014). The clinical relevance of dry powder inhaler performance for drug delivery. Respir. Med..

